# Informal Caregivers’ Use of Internet-Based Health Resources: An Analysis of the Health Information National Trends Survey

**DOI:** 10.2196/11051

**Published:** 2018-12-18

**Authors:** Kelly M Shaffer, Philip I Chow, Wendy F Cohn, Karen S Ingersoll, Lee M Ritterband

**Affiliations:** 1 Department of Psychiatry and Behavioral Sciences Memorial Sloan Kettering Cancer Center New York, NY United States; 2 Center for Behavioral Health & Technology Department of Psychiatry and Neurobehavioral Sciences University of Virginia School of Medicine Charlottesville, VA United States; 3 Department of Public Health Sciences University of Virginia School of Medicine Charlottesville, VA United States

**Keywords:** caregivers, health education, internet, information dissemination, mobile phone, uncompensated care

## Abstract

**Background:**

Informal caregivers express strong interest in technology innovations to help them in their caregiving role; however, divides across sociodemographic characteristics in internet and technology access may preclude the most vulnerable caregivers from accessing such resources.

**Objective:**

This study aims to examine caregivers’ internet use, both generally and for seeking health-related information, and whether usage differs as a function of caregivers’ characteristics.

**Methods:**

Data were analyzed from the Health Information National Trends Survey 5 Cycle 1. Participants were included in analyses if they self-identified as providing uncompensated care to a close individual. Caregivers reported internet use factors, age, education, rurality, general health, distress, and objective caregiving burden. We used chi-square tests of independence with jackknife variance estimation to compare whether internet use factors differed by caregivers’ characteristics.

**Results:**

A total of 77.5% (303/391) caregivers surveyed reported ever using the internet. Of internet users, 88.1% (267/303) accessed from a home computer and 83.2% (252/303) from a mobile device. Most caregivers accessed health information for themselves (286/391, 73.1%) or others (264/391, 67.5%); fewer communicated with a doctor over the Web (148/391, 37.9%) or had a wellness app (171/391, 43.7%). Caregivers reporting younger age, more education, and good health were more likely to endorse any of these activities. Furthermore, two-thirds of caregivers (258/391, 66.0%) endorsed trust in health information from the internet.

**Conclusions:**

Computers and mobile devices are practical platforms for disseminating caregiving-related information and supportive services to informal caregivers; these modalities may, however, have a more limited reach to caregivers who are older, have less education, and are in poorer health.

## Introduction

Over 42 million Americans provided unpaid care to a loved one with serious illness in 2015 [1]; these informal caregivers provide practical, medical, and emotional assistance to people with illness, often with little preparation [2,3] and risking the decline of their own mental and physical health [4-7]. Caregivers see technology (eg, computers, smartphones, and tablets) as having the marked potential to assist them in their caregiver role by offering conveniently accessible information, support, and organizational tools [8]. Indeed, caregivers are more likely than noncaregivers to have internet access and access health-related information over the Web [9]. As such, psychosocial researchers and caregiver advocates are also turning to internet-based programs as resources for this population, given caregivers’ high barriers to accessing traditional in-person supportive care [8,10-14]. Although inequalities are narrowing, prevailing disparities in internet and technology access suggest that digital tools and interventions may not be equivalently accessible by all caregivers [15,16]. Americans aged ≥65 years, with high school education or less, and living in rural areas are both less likely to report home internet access or own a smartphone [15] and more likely to experience poor health outcomes from caregiving [17-19]. These disparities suggest that some of the most vulnerable caregivers may be least likely to benefit from internet- and technology-delivered resources, warranting further investigation.

Informal caregivers are a high-risk population for poor mental and physical health outcomes. Compared with the general population, caregivers report higher rates of depression and anxiety [20-22], have poorer healthy lifestyle behaviors, such as diet, exercise, and sleep [23-25], and exhibit premature physical health decline [4,6,7]. Such discrepancies are likely related, in part, to the high stresses of caregiving. Caregivers report spending 24 hours per week on average on caregiving tasks [1], although demands can be markedly greater depending on the care recipient disease severity; caregivers for people with Alzheimer’s disease are estimated to spend an average of 6 hours per day on caregiving [26], and cancer caregivers have described feeling “on-call” 24 hours per day [27]. Caregiving includes tasks such as assisting a loved one with activities of daily living and medical tasks, distributing information to family and friends, and navigating health care decisions [1]. Although in-person psychosocial interventions and educational tools have been effective in improving caregivers’ well-being and role mastery [28-30], these interventions experience low enrollment, high dropout, and limited reach to caregivers of lower socioeconomic strata.

Disseminating caregiving resources through the internet may lower the barrier to entry and increase accessibility for caregivers by affording caregivers timely and convenient access to resources. Among the general US population, the internet fills an important gap in meeting health information needs; individuals with the highest barriers to health care access (ie, those who are traditionally underserved) are most likely to search for health-related information over the Web [31]. Over 90% of caregivers express interest in using internet- and technology-based tools to assist them in their caregiving role, with a particular interest in tools to help with managing medication refill and adherence, medical appointments, and emotional strain from caregiving [8]. While the acceptability of these tools seems apparent, <10% of caregivers report actually using any such tools that are currently available over the Web [8], raising questions regarding the extent to which all caregivers will be able to access and benefit equally from such internet- and technology-delivered resources. Such resources may range in complexity from static informational websites to websites tailored based on user input, multicomponent programs and apps [13,14]. The uptake of these varying resources is influenced by caregivers’ digital literacy (eg, personal capacity for technology use, including computer proficiency), accessibility of technology, caregiving needs, and stress [32]. Understanding how caregivers’ sociodemographic factors may interact with their natural propensity to use different internet resources will help identify caregivers for whom internet-based tools may have the greatest, and least, reach.

Therefore, this study examines data from a large, nationally representative sample to better understand the potential reach of internet- and technology-delivered caregiving resources. The primary aims of this investigation are to characterize internet use among informal caregivers and examine whether internet usage differs according to certain sociodemographic, health, and caregiving-related factors. First, we characterize caregivers’ self-reported internet use: both generally—whether they ever reported using the internet for any reason—and specifically for seeking health information. Next, we investigate whether such internet use differs according to factors potentially associated with disparities, namely caregivers’ age, educational attainment, rurality, general health, distress, and caregiving burden.

## Methods

### Study Design and Participants

This is a cross-sectional study using data from the Health Information National Trends Survey (HINTS, 2017) [33] Version 5, Cycle 1, a nationally representative survey of civilian, noninstitutionalized adults aged ≥18 years in the United States. This survey is conducted by the National Cancer Institute (NCI) and assesses Americans’ access to and use of health information. The HINTS was reviewed and approved by the Institutional Review Boards of the NCI Special Studies and the main contractor (Westat, Inc). Surveys were distributed by mail between January 2017 and May 2017. Of 13,360 surveys mailed, 3285 participants returned surveys (response rate, 32.4%). Additional methodology details are provided in the Methodology Report [34].

This analysis exclusively reports data from 391 participants who self-identified as a caregiver for an adult by answering affirmatively to the item “Are you currently caring for or making health care decisions for someone with a medical, behavioral, disability, or some other condition?” Caregivers could indicate that they were providing care to a spouse, parent, another family member, or another close individual; caregivers could select multiple responses to indicate they provided care for multiple individuals. Informal caregiving surveys typically focus on informal care to adults, given the anomalous, age-discordant nature of this caregiving [1,8]. Informal caregiving for a child is typically assessed with a denotation of atypical care provision, for example, “this kind of care is more than the normal care required for a child” [1]; no such specification was provided in the HINTS survey. As such, for this analysis, participants exclusively reporting caring for a child were not included (n=189); however, individuals endorsing care for both a child and an adult were included (n=64).

### Measures

The specific wording of all measures reported in this study is available on the Web [35].

#### Internet Use Variables

Outcomes of interest were assessed by the following single-item questions.

##### Internet Use

Internet use was ascertained by participants responding to “Do you ever go on-line to access the Internet or World Wide Web, or to send and receive e-mail?” Response options were *Yes* or *No*.

##### Internet Use by Home Computer and/or Mobile Device

Those participants answering affirmatively to internet use were then asked, “How often do you access the Internet through each of the following…Computer at home?…On a mobile device (cell phone/smartphone/tablet)?” Response options were dichotomized to *Daily* and *Sometimes* versus *Never* and *Not applicable* for each item separately.

##### Wellness Mobile Apps

All participants were asked, “On your tablet or smartphone, do you have any ‘apps’ related to health and wellness?” Response options were *Yes*, *No*, *Don’t know*, or *Do not have a tablet or smartphone*. Participants who indicated that they did not have a tablet or smartphone were excluded from analyses with this variable (50/391, 12.8%). Remaining responses were dichotomized to *Yes* versus *No* and *Don’t know*.

##### Electronic Access of Health Information for Self and/or Someone Else and Contact With Doctor

All participants were asked, “In the past 12 months, have you used a computer, smartphone, or other electronic means to do any of the following…Looked for health or medical information for yourself?…Looked for health or medical information for someone else?…Used e-mail or the Internet to communicate with a doctor or a doctor’s office?” Response options were *Yes* or *No* for each item.

##### Trust in Health Information From the Internet

All participants were asked, “In general, how much would you trust information about health or medical topics from each of the following…Internet?” Response options were dichotomized to *Not at all* and *A little* versus *Some* and *A lot*.

#### Caregiver Characteristics

Dichotomized variables for caregivers’ age (*18-64* vs ≥*65*), level of education (*high school or less* vs *some college or more*), rurality (*metropolitan* vs *nonmetropolitan*), general health (self-reported as *excellent*, *very good*, or *good* vs *fair* or *poor*), distress [Patient Health Questionnaire (PHQ)-4 total score of ≤*2* vs ≥*3*], and caregiving burden (time spent providing care *<5 hours/week* vs ≥*5 hours/week*) were examined for their relations to the internet use variables.

Age was dichotomized at 65 years (as reported previously [1]). Rurality was determined according to the National Center for Health Statistics Urban–Rural Classification Scheme for Counties, which was dichotomized to capture metropolitan (large metro to small metro) versus nonmetropolitan (micropolitan and noncore) counties of participants’ residence. Distress was assessed by the PHQ-4 [36], which includes 2 items assessing anxiety and 2 items assessing depressed mood. Items are rated on a Likert scale score from 0 (*Not at all*) to 3 (*Nearly every day*). Higher sum scores indicate greater overall distress, with scores of ≥3 demarking clinically meaningful symptoms. The PHQ-4 has demonstrated strong reliability and validity for measuring depression and anxiety in the general population [36]. Caregivers’ reported hours spent caregiving per week (response options included “less than 5 hours per week,” “5-14 hours per week,” “15-20 hours per week,” “21-34 hours per week,” and “35 or more hours per week”) represented an approximate measure of the objective caregiving burden.

### Analytical Strategy

#### Principal analyses

Descriptive statistics (means with SDs or frequencies, as appropriate) for study variables were conducted using SPSS version 25 (Tables 1 and 2). Research aims were addressed by weighted chi-square tests of independence that tested whether caregivers’ characteristics related to internet use variables. Data were analyzed using SAS 9.4 SURVEYFREQ procedures to account for the complex sampling design of the HINTS survey. Analyses were weighted using the full-sample weights provided in the public use datasets, yielding nationally representative population estimates. In addition, the jackknife variance estimation with repeated replications was used to estimate SEs, which reduces bias and, therefore, type I error. These procedures are in accordance with published HINTS analysis recommendations [37]. Furthermore, alpha of .05 was used to determine significance for all tests.

#### Post-hoc analyses

Upon completion of principal analyses, we noted that 39 caregivers (10.0%) reported having accessed information or communicating with a doctor by internet-based or other electronic methods in the past year (ie, Information: Self, Information: Other, and/or Talk with Doctor=Yes), yet denied ever having used the internet (ie, Use Internet=No); this discrepancy suggests that some users may have incorrectly characterized themselves as noninternet users. To increase confidence that the planned analyses captured all caregivers who used the internet, a variable was computed that combined anyone who indicated affirmatively to any of the items—Information: Self, Information: Other, Talk with Doctor, and/or Use Internet. Then, weighted chi-square tests of independence were repeated, comparing the proportions of caregivers endorsing this expanded Use Internet variable by age, education, rurality, distress, and caregiving burden.

## Results

### Principal Results

Table 1 presents sample characteristics for participating caregivers’ sociodemographic, health, and caregiving factors. Caregivers’ average age was 58 (range: 24-101) years, 55.0% (215/391) of the sample self-identified as non-Hispanic white, 64.5% (252/391) identified as female, and 21.0% (82/391) reported having children living in their home. Caregivers were most commonly (181/391, 46.3%) caring for a parent. Aging-related health issues (165/391, 42.2%) were most commonly reported as a reason that the care recipient required assistance. Most caregivers reported some college education or more (293/391, 74.9%), and lived in a metropolitan county (330/391, 84.4%). Most caregivers endorsed good health or better (319/391, 81.6%) and minimal distress (251/391, 64.2%). Caregivers most commonly reported spending ≥5 hours/week providing care to the care recipient(s) (228/391, 58.3%).

### Aim 1: Characterize Internet Use Among Caregivers

Table 2 provides detailed descriptions of participating caregivers’ internet use variables. Most caregivers indicated that they had used the internet (303/391, 77.5%); of these, most reported that they accessed the internet from a computer at home (267/391, 88.1%) and/or a mobile device (252/391, 83.2%). Among those with a tablet or smartphone, a similar percentage of caregivers endorsed having a wellness mobile app (171/391, 43.7%) versus not having one (165/391, 42.2%). Most caregivers reported having used electronic means to access health information for themselves (286/391, 73.1%) or someone else (264/391, 67.5%) in the past year; a few reported having communicated with a doctor using the internet or email (148/391, 37.9%). Furthermore, two-thirds of caregivers (258/391, 66.0%) endorsed mostly trusting medical information from the internet.

### Aim 2: Examine Internet Use Across Caregiver Characteristics

Table 3 presents results for weighted chi-square tests comparing internet use generally (Use Internet) and internet use by home computer (Use Internet: Home) or mobile device (Use Internet: Mobile). Older age, less education, and metropolitan residence were associated with a lower likelihood of endorsing internet use. Among those caregivers endorsing internet use, worse general health was associated with a lower likelihood of accessing the internet from home computer, and older age was associated with a lower likelihood of accessing the internet from a mobile device.

Table 4 presents results for weighted chi-square tests comparing a wellness mobile app use (Wellness App) and comparing electronic health information access for one’s self (Information: Self) or another person (Information: Other). Among those owning a tablet or smartphone, older age was associated with a lower likelihood of having downloaded a wellness app. Only older age was associated with a lower likelihood of using the internet to access information for one’s self. Furthermore, older age and less education were associated with a lower likelihood of using the internet to access information for someone else.

Table 5 presents results for weighted chi-square tests, electronic communication with a doctor (Talk with doctor), and trust in health information from the internet (Trust internet information). Older age and worse general health were associated with a lower likelihood of electronic communication with a doctor. Worse general health was associated with less trust in health information from the internet.

**Table 1:** Sample description (N=391).

Characteristics			Value^a^
**Sample characteristics**			
	Age, mean (SD)		58.3 (13.9)
	Gender (Female), n (%)		252 (64.5)
	**Race or ethnicity, n (%)**		
		Non-Hispanic White	215 (55.0)
		Non-Hispanic Black or African American	55 (14.1)
		Hispanic	48 (12.3)
		Non-Hispanic Asian	22 (5.6)
		Non-Hispanic other	18 (4.6)
	**Relationship to care recipient^b^, n (%)**		
		Spouse	147 (37.6)
		Child	181 (46.3)
		Other family	77 (19.1)
		Friend or other	38 (9.7)
	**Care recipient condition^c^, n (%)**		
		Cancer	65 (16.6)
		Alzheimer’s or dementia or cognitive impairment	132 (33.8)
		Aging	165 (42.2)
	**Children in home, n (%)**		
		0	279 (71.4)
		1-2	64 (16.4)
		≥3	18 (4.6)
**Caregiver characteristics examined for impact**			
	**Age group, n (%)**		
		18-64	251 (64.2)
		≥65	123 (31.5)
	**Level of education, n (%)**		
		High school or less	86 (22.0)
		Some college or more	293 (74.9)
	**Rurality, n (%)**		
		Metropolitan	330 (84.4)
		Nonmetropolitan	61 (15.6)
	**General health, n (%)**		
		Excellent, Very good, Good	319 (81.6)
		Fair, poor	68 (17.4)
	**Distress (Patient Health Questionnaire-4), n (%)**		
		Minimal symptoms (≤2)	251 (64.2)
		Mild symptoms or more (≥3)	128 (32.7)
	**Caregiving burden, n (%)**		
		<5 hours/week	136 (34.8)
		≥5 hours/week	228 (58.3)

^a^Numbers may not total to N where data are missing.

^b^Caregivers may provide care to >1 relative.

^c^Care may be provided to care recipients for multiple conditions.

**Table 2:** Internet usage and electronic health information access among caregivers (N=391).

Questions					n^a^ (%)	
**Do you ever go online to access the internet or World Wide Web, or to send and receive email?**						
	No				88 (22.5)	
	**Yes**				303 (77.5)	
		**If yes, how often do you access the internet by:^b^**				
			**A computer at home?**			
				Daily or Sometimes		267 (88.1)^c^
				Never or Nonapplicable		31 (10.2)^c^
			**A mobile device?**			
				Daily or Sometimes		252 (83.2)^c^
				Never or Nonapplicable		38 (12.5)^c^
**On your tablet or smartphone, do you have any “apps” related to health and wellness?^d^**						
	Yes				171 (43.7)	
	No or Don’t know				165 (42.2)	
**In the past 12 months, have you used a computer, smartphone, or other electronic means to:**						
	**Look for health/medical info for yourself?**					
		Yes				286 (73.1)
		No				105 (26.9)
	**Look for health/medical info for someone else?**					
		Yes				264 (67.5)
		No				126 (32.2)
	**Communicate with a doctor using email/internet?**					
		Yes				148 (37.9)
		No				242 (61.9)
**In general, how much would you trust information about health or medical topics from the internet?**						
	A lot or Some				258 (66.0)	
	A little or Not at All				108 (27.6)	

^a^Numbers may not total to N=391 where data are missing.

^b^Only caregivers indicating that they ever used the internet were eligible to respond.

^c^Percentage of those endorsing “Yes” to internet Use (ie, N=303).

^d^Caregivers who reported not having a tablet or smartphone were excluded.

**Table 3:** Differences in caregivers’ internet usage by their characteristics.

Caregivers’ characteristics			Use Internet				Use Internet: Home			Use Internet: Mobile			
			Yes, n (%)^a^	No, n (%)	*F*^b^		Yes, n (%)	No, n (%)	*F*		Yes, n (%)	No, n (%)	*F*
**Age (years)**					*8.80* ^c^				0.34				*10.96*
	18-64		212 (86.3)	39 (13.7)			192 (91.7)	18 (8.3)			193 (96.7)	16 (3.3)	
	≥65		83 (59.6)	40 (40.4)			70 (87.2)	11 (12.8)			52 (68.8)	22 (31.2)	
**Education**					*5.44*				1.34				3.1
	≤High school		53 (68.3)	33 (31.7)			45 (84.9)	7 (15.1)			39 (84.4)	11 (15.6)	
	≥Some college		247 (83.8)	46 (16.2)			220 (92.8)	23 (7.2)			211 (95.1)	26 (4.9)	
**Rurality**					*7.07*				0.85				1.24
	Metropolitan		253 (75.6)	77 (24.4)			225 (92.0)	25 (8.0)			215 (93.5)	28 (6.5)	
	Nonmetropolitan		50 (90.5)	11 (9.5)			42 (85.3)	6 (14.7)			37 (85.8)	10 (14.2)	
**General health**					2.54				*5.35*				2.16
	≥Good		264 (82.6)	55 (17.4)			237 (94.9)	22 (5.1)			222 (94.0)	30 (6.0)	
	≤Fair		39 (63.9)	29 (36.1)			30 (67.0)	9 (33.0)			30 (81.5)	8 (18.5)	
**Distress (PHQ-4^d^)**					1.36				0				0.05
	Minimal (≤2)		199 (75.5)	52 (24.5)			175 (90.7)	21 (9.3)			171 (92.5)	20 (7.5)	
	Mild or more (≥3)		97 (83.0)	31 (17.0)			86 (90.7)	9 (9.3)			77 (91.7)	16 (8.3)	
**Caregiving burden**					1.6				0				2.81
	<5 hours/week		114 (84.8)	22 (15.2)			99 (90.7)	13 (9.3)			97 (95.4)	11 (4.6)	
	≥5 hours/week		177 (78.0)	51 (22.0)			158 (91.1)	16 (8.9)			144 (90.2)	26 (9.8)	

^a^Numbers may not match with totals reported in Table 2 where data are missing, and percentages are weighted using jackknife weighting.

^b^All chi-square tests have degree of freedom (1, 49)—*F*>4.04, *P*<.05; *F*>7.19, *P*<.01; *F*>12.37, *P*<.001.

^c^Comparisons with *P*<.05 are presented in italics for reference.

^d^PHQ-4: Patient Health Questionnaire-4.

**Table 4:** Differences in caregivers’ use of wellness apps and access to electronic health information by their characteristics.

Caregivers’ characteristics		Wellness App			Information: Self			Information: Other		
		Yes, n (%)^a^	No or don’t know, n (%)	*F* ^b^	Yes, n (%)	No, n (%)	*F*	Yes, n (%)	No, n (%)	*F*
**Age (years)**				*4.04* ^c^			*9.89*			*13.82*
	18-64	127 (59.4)	106 (40.6)		198 (80.2)	53 (19.8)		191 (79.8)	60 (20.2)	
	≥65	36 (43.5)	57 (56.5)		76 (56.7)	47 (43.3)		64 (47.0)	59 (53.0)	
**Education**				1.63			2.83			*6.54*
	≤High school	32 (48.7)	37 (51.3)		51 (65.0)	35 (35.0)		43 (56.1)	43 (43.9)	
	≥Some college	138 (60.9)	126 (39.1)		229 (79.5)	64 (20.5)		217 (78.6)	76 (21.4)	
**Rurality**				1.98			1.24			0.09
	Metropolitan	150 (59.6)	135 (40.4)		245 (76.2)	85 (23.8)		225 (71.5)	104 (28.5)	
	Nonmetropolitan	21 (44.2)	30 (55.8)		41 (63.9)	20 (36.1)		39 (68.7)	22 (31.3)	
**General health**				3.72			0.55			1.05
	≥Good	149 (60.6)	131 (39.4)		239 (76.8)	80 (23.2)		225 (73.8)	94 (26.2)	
	≤Fair	22 (43.1)	32 (56.9)		47 (69.9)	21 (30.1)		38 (63.4)	29 (36.6)	
**Distress (PHQ-4^d^)**				0.11			0.12			0.30
	Minimal (≤2)	114 (59.0)	104 (41.0)		186 (73.5)	65 (26.5)		175 (69.3)	75 (30.7)	
	Mild or more (≥3)	55 (56.5)	54 (43.5)		92 (75.9)	36 (24.1)		46 (73.4)	82 (26.6)	
**Caregiving burden**				0.03			1.26			0.26
	<5 hours/week	62 (58.1)	62 (41.9)		106 (81.1)	30 (18.9)		96 (75.1)	40 (24.9)	
	≥5 hours/week	104 (59.5)	87 (40.5)		168 (73.4)	60 (26.6)		69 (71.9)	158 (28.1)	

^a^Numbers may not match with totals reported in Table 2 where data are missing, and percentages are weighted using jackknife weighting.

^b^All chi-square tests have degree of freedom (1, 49)—*F*>4.04, *P*<.05; *F*>7.19, *P*<.01; *F*>12.37, *P*<.001.

^c^Comparisons with *P*<.05 are presented in italics for reference.

^d^PHQ-4: Patient Health Questionnaire-4.

**Table 5:** Differences in caregivers’ communication with doctors and trust in information from the internet by their characteristics.

Caregivers’ characteristics		Talk with doctor			Trust internet information			
		Yes, n (%)^a^	No, n (%)	*F* ^b^	A lot or Some, n (%)	A little or none, n (%)	*F*	
**Age (years)**				*9.64* ^c^			4.01	
	18-64	110 (45.9)	140 (54.1)		180 (73.3)	62 (26.7)		
	≥65	35 (24.8)	88 (75.2)		72 (55.8)	38 (44.2)		
**Education**				2.19				
	≤High school	25 (32.7)	61 (67.3)		47 (60.0)	30 (40.0)		
	≥Some college	123 (44.1)	169 (55.9)		209 (72.3)	72 (27.7)		
**Rurality**				0.03			0.09	
	Metropolitan	126 (39.4)	203 (60.6)		219 (68.1)	87 (31.9)		
	Nonmetropolitan	22 (41.1)	39 (58.9)		39 (65.3)	21 (34.7)		
**General health**				*6.37*			*6.77*	
	≥Good	132 (44.3)	187 (55.7)		225 (73.9)	77 (26.1)		
	≤Fair	16 (21.6)	51 (78.4)		31 (42.7)	29 (57.3)		
**Distress (PHQ-4^d^)**				0.03			0.07	
	Minimal (≤2)	103 (39.6)	148 (60.4)		170 (68.3)	66 (31.7)		
	Mild or more (≥3)	43 (40.9)	84 (59.1)		80 (66.3)	41 (33.7)		
**Caregiving burden**				2.28			1.85	
	<5 hours/week	63 (48.9)	73 (51.1)		99 (76.5)	29 (23.5)		
	≥5 hours/week	81 (37.0)	146 (63.0)		150 (66.0)	66 (34.0)		

^a^Numbers may not match with totals reported in Table 2 where data are missing, and percentages are weighted using jackknife weighting.

^b^All chi-square tests have degree of freedom (1, 49)—*F*>4.04, *P*<.05; *F*>7.19, *P*<.01; *F*>12.37, *P*<.001.

^c^Comparisons with *P*<.05 are presented in italics for reference.

^d^PHQ-4: Patient Health Questionnaire-4.

### Post-Hoc Analyses

Weighted chi-square tests of independence were repeated for the expanded Use Internet item (ie, answering affirmatively to any of the items: Information: Self, Information: Other, Talk with Doctor, and/or Use Internet) that accounted for caregivers’ potential misidentification as noninternet users. Results indicated a comparable pattern of findings between the original Use Internet and expanded Use Internet variables—older age (*F*_1,49_=5.02, *P*=.03) and lower education (*F*_1,49_=4.37, *P*=.04) were associated with lower reported internet use; general health (*F*_1,49_=2.42, *P*=.25), distress (*F*_1,49_=1.68, *P*=.20), and caregiving burden (*F*_1,49_=3.74, *P*=.06) were not associated with the reported internet use. The only discrepant finding for the expanded Use Internet variable compared with the original Use Internet variable was that rurality was no longer significantly associated with internet use (*F*_1,49_=2.42, *P*=.13).

## Discussion

### Principal Findings

Using data from a US nationally representative survey, this paper presents novel evidence regarding how internet use, both generally and specifically for accessing health-related information, differs among informal caregivers according to certain key sociodemographic, health, and caregiving-related factors. Overall, the internet use, both by home computers and mobile devices, was common among caregivers, as was accessing health information for themselves and other people through the internet. The proportion of caregivers in this study who endorsed ever using the internet (303/391, 77.5%) was lower than prior estimates of internet use among the general population (89%) [15] or among caregivers (86%-92%) [8,9], although adjusting to include individuals who indicated any electronic access of health-related communication or information resulted in more comparable rates (342/391, 87.5%). The use of wellness mobile apps and communicating with a doctor’s office by the internet were less common among caregivers. Older age, lower educational attainment, and poorer general health were associated with less frequent internet activity, as has been found among the general population [15]. Furthermore, rurality, distress, and caregiving burden were generally not related to internet activity.

From this survey data, caregivers aged ≥65 years tended to report lower overall internet use, with an estimated 40.4% of the population of older caregivers identifying as noninternet users, compared with 13.7% of caregivers under the age of 65 years. Among internet users, older caregivers did not differ from younger caregivers in terms of home computer internet access but did endorse an approximately 10-fold lower likelihood to access the internet by a mobile device. Growth trends in mobile phone use are highest among older adults [38], however, suggesting the potential longer-term utility of mobile phones as a tool for enhancing older caregivers’ well-being [13,39]. Fewer older caregivers reported having a wellness “app” downloaded, searching for health- or medical-related information, and communicating with a doctor’s office using the internet. These findings regarding the disparity across the types of internet use by age are consistent with prior studies of both the general population [15,40] and caregivers [8], in which older adults are less likely to use the internet either in general or for health-related purposes. Low-barrier caregiving-related resources, such as those delivered by the internet, may be especially important for older caregivers, being particularly vulnerable owing to frequently having less help with caregiving, more medical problems of their own, and more barriers to accessing traditional in-person resources [1,17,18]. Importantly, older caregivers report being equally as interested in internet-based resources to assist them in their caregiving role as were younger caregivers [8], meaning older adults’ lower internet use should not necessarily be interpreted as a lack of demand among this group. Interventions designed to enhance older caregivers’ internet skills and digital literacy may help increase reach and benefit of internet- and technology-based health resources to this rapidly growing and especially vulnerable subset of informal caregivers (for review of similar programs, see Ref. [41]).

Caregivers reporting fair or poor overall health, another vulnerable caregiving population, were equally as likely to report internet use as caregivers reporting good or better health, yet reported 6-fold lower access from a home computer. In terms of dissemination of caregiver resources by the internet, ensuring that Web-based resources are compatible with both computer and mobile browsers will ensure the broadest reach of such resources. Many programs developed for caregivers, however, are not cross-platform appropriate (eg, disseminated through a native app vs a mobile-friendly website) [10-14], which would limit reach across caregivers. In addition, caregivers with poorer health were less likely to have communicated with a doctor or doctor’s office through the internet and were twice as likely to distrust health-related information from the internet, despite being equally likely to access health information online. Notably, the HINTS survey did not qualify the item assessing trust in health information from the internet in terms of website reputability. It may be the case that caregivers in poorer health have had more experience finding “bad” information about health conditions online, thus expressing less trust in this modality. Overall, establishing reputability of internet- and technology-based caregiver resources, by ensuring the accuracy of information and creating a professional and appealing user experience, will be important to earn caregivers’ trust and uptake [42].

Contrary to expectations, caregivers living in a rural area were more likely to report internet use than those in metropolitan areas, although this finding did not hold in post-hoc analyses when broadening the internet use variable to account for those potentially misidentifying as noninternet users. The weighted percentage of internet use among the current sample of rural caregivers (90.5%) was substantively higher than prior nationally representative samples of adults living in rural areas (78%) [15], although the unweighted percentage (50/61, 82.0%) was more comparable. Given caregivers in rural areas have less access to traditional in-person health care resources, internet- and technology-based resources may help meet supportive care demand for these caregivers [31]. In addition, caregivers’ internet activity did not differ according to caregivers’ level of distress or caregiving burden. These findings contrast with previous qualitative findings among dementia caregivers, who had described stress and burden from caregiving as factors that interfered with their use of an internet-based intervention [32]. Further research into how caregiving stresses—both objective (eg, time and care tasks) and subjective (eg, perceived burden and impact of caregiving)—affect the uptake and use of internet-based resources will help to ensure the greatest reach and utility of these resources for caregivers who most need them.

### Strengths, Limitations, and Future Directions

This study reports findings from a cross-sectional survey that was designed to capture the attitudes of cancer-related health information from a representative sample of the general US adult population. As such, this survey was not designed to address research questions for this study specifically nor was sampling specifically targeted to family caregivers. Hence, this survey did not ascertain subjective caregiving burden or specific caregiving responsibilities, which may affect caregivers’ motivation or need to access health information by the internet. In addition, it was not ascertained whether caregivers were utilizing the internet specifically to assist them in their caregiving role; however, this has been previously well-described [8], and this study extends the prior research to better understand what subsets of caregivers may be less likely to benefit from caregiver resources disseminated through the internet. Moreover, it should be noted that we analyzed a subset of the overall HINTS 5 cycle 1 sample (N=3285), namely those identifying as informal caregivers to adults (n=391), who were not representatively sampled among the national population of informal caregivers. This may reduce the reliability and generalizability of findings; however, robust analytical techniques that incorporate the complex survey weighting mitigates these concerns. Furthermore, these analytical techniques provide population estimates that are likely to be more representative than most prior research with caregivers, which has tended to disproportionately capture socioeconomically advantaged and non-Hispanic white individuals.

Ultimately, internet- and technology-based education, communication, and intervention hold significant promise to help caregivers be more active and connected participants in their care recipients’, and their own, health care [12,39]. Although findings here suggest older caregivers, those with lower educational attainment, and those in poorer health are less likely to be using existing internet resources, it is not known why these individuals report less frequent use. Potential reasons explaining these discrepancies may include limited knowledge of how to use technology, privacy concerns, costs of technology, lack of awareness of available internet resources, perception that the effort of using such resources would not outweigh potential benefits, or preferring more traditional resources, like print information or communicating with doctors by phone or at clinic appointments. Moreover, little is known about how such resources fit into caregivers’ day-to-day lives or complement existing health care delivery systems [12], factors that may relate to caregivers’ long-term use of and benefit from such tools. Continued research to understand caregivers’ evolving needs, preferences for meeting those needs, and how needs and preferences differ among caregivers is necessary to ensure all caregivers may optimally benefit from internet- and technology-based caregiving resources.

### Conclusions

Most caregivers are internet users, both for general purposes and specifically to access health information. Caregivers over the age of 65 years, those with a high school education or less, and those reporting poor health report less internet activity, suggesting these vulnerable caregivers may not be equally benefited by internet- and technology-based caregiver resources without careful consideration of factors that might facilitate their use. In addition, caregivers’ level of rurality, distress, and caregiving burden did not relate to their internet activity, suggesting these factors are not likely to impede caregivers’ access to internet- and technology-based resources. Overall, internet- and technology-based resources to support informal caregivers are likely to have significant reach, yet close attention to the accessibility of such resources by the most vulnerable caregivers must be paid to ensure equal benefit.

## Abbreviations

HINTS: Health Information National Trends Survey
NCI: National Cancer Institute

## References

[1] National Association for Caregiving, AARP (2015). Caregiving in the US.

[2] Havyer RD, van Ryn M, Wilson PM, Griffin JM (2017). The effect of routine training on the self-efficacy of informal caregivers of colorectal cancer patients. Support Care Cancer.

[3] van Ryn M, Sanders S, Kahn K, van Houtven C, Griffin JM, Martin M, Atienza AA, Phelan S, Finstad D, Rowland J (2011). Objective burden, resources, and other stressors among informal cancer caregivers: a hidden quality issue?. Psychooncology.

[4] Ji J, Zöller B, Sundquist K, Sundquist J (2012). Increased risks of coronary heart disease and stroke among spousal caregivers of cancer patients. Circulation.

[5] Pinquart M, Sörensen S (2003). Differences between caregivers and noncaregivers in psychological health and physical health: a meta-analysis. Psychol Aging.

[6] Shaffer KM, Kim Y, Carver CS, Cannady RS (2017). Effects of caregiving status and changes in depressive symptoms on development of physical morbidity among long-term cancer caregivers. Health Psychol.

[7] Buyck J, Ankri J, Dugravot A, Bonnaud S, Nabi H, Kivimäki M, Singh-Manoux A (2013). Informal caregiving and the risk for coronary heart disease: the Whitehall II study. J Gerontol A Biol Sci Med Sci.

[8] AARP, Project Catalyst (2016). Caregivers and Technology: What they Want and Need.

[9] Fox S, Duggan M, Purcell K (2013). Pew Research Center.

[10] Badr H, Carmack CL, Diefenbach MA (2015). Psychosocial interventions for patients and caregivers in the age of new communication technologies: opportunities and challenges in cancer care. J Health Commun.

[11] Boots LMM, de Vugt ME, van Knippenberg RJM, Kempen GIJM, Verhey FRJ (2014). A systematic review of Internet-based supportive interventions for caregivers of patients with dementia. Int J Geriatr Psychiatry.

[12] Dyer E, Kansagara D, McInnes D, Freeman M, Woods S (2012). Mobile applications and Internet-based approaches for supporting non-professional caregivers: a systematic review. Department of Veterans Affairs, Washington (DC).

[13] Grossman MR, Zak DK, Zelinski EM (2018). Mobile Apps for Caregivers of Older Adults: Quantitative Content Analysis. JMIR Mhealth Uhealth.

[14] Wasilewski MB, Stinson JN, Cameron JI (2017). Web-based health interventions for family caregivers of elderly individuals: A Scoping Review. Int J Med Inform.

[15] (2018). Pew Research Center.

[16] Viswanath K (2006). Public communications and its role in reducing and eliminating health disparities. Examining the health disparities research plan of the national institutes of health: unfinished business. Institute of Medicine.

[17] Pinquart M, Sörensen S (2007). Correlates of physical health of informal caregivers: a meta-analysis. J Gerontol B Psychol Sci Soc Sci.

[18] Richardson TJ, Lee SJ, Berg-Weger M, Grossberg GT (2013). Caregiver health: health of caregivers of Alzheimer's and other dementia patients. Curr Psychiatry Rep.

[19] Butler SS, Turner W, Kaye LW, Ruffin L, Downey R (2005). Depression and caregiver burden among rural elder caregivers. J Gerontol Soc Work.

[20] Cochrane JJ, Goering PN, Rogers JM (1997). The mental health of informal caregivers in Ontario: an epidemiological survey. Am J Public Health.

[21] Lambert SD, Girgis A, Lecathelinais C, Stacey F (2013). Walking a mile in their shoes: anxiety and depression among partners and caregivers of cancer survivors at 6 and 12 months post-diagnosis. Support Care Cancer.

[22] Price MA, Butow PN, Costa DSJ, King MT, Aldridge LJ, Fardell JE, DeFazio A, Webb PM, Australian Ovarian Cancer Study Group, Australian Ovarian Cancer Study Group Quality of Life Study Investigators (2010). Prevalence and predictors of anxiety and depression in women with invasive ovarian cancer and their caregivers. Med J Aust.

[23] Chassin L, Macy JT, Seo D, Presson CC, Sherman SJ (2010). The Association between Membership in the Sandwich Generation and Health Behaviors: A Longitudinal Study. J Appl Dev Psychol.

[24] Hoffman GJ, Lee J, Mendez-Luck CA (2012). Health behaviors among Baby Boomer informal caregivers. Gerontologist.

[25] Morris BA, Thorndike FP, Ritterband LM, Glozier N, Dunn J, Chambers SK (2015). Sleep disturbance in cancer patients and caregivers who contact telephone-based help services. Support Care Cancer.

[26] Wimo A, Gauthier S, Prince M (2018). Alzheimer's Disease International (ADI).

[27] Kim Y, Spillers RL (2010). Quality of life of family caregivers at 2 years after a relative's cancer diagnosis. Psychooncology.

[28] Applebaum AJ, Breitbart W (2013). Care for the cancer caregiver: a systematic review. Palliat Support Care.

[29] Romito F, Goldzweig G, Cormio C, Hagedoorn M, Andersen BL (2013). Informal caregiving for cancer patients. Cancer.

[30] Northouse L, Williams A, Given B, McCorkle R (2012). Psychosocial care for family caregivers of patients with cancer. J Clin Oncol.

[31] Bhandari N, Shi Y, Jung K (2014). Seeking health information online: does limited healthcare access matter?. J Am Med Inform Assoc.

[32] Chiu TML, Eysenbach G (2011). Theorizing the health service usage behavior of family caregivers: a qualitative study of an internet-based intervention. Int J Med Inform.

[33] Nelson DE, Kreps GL, Hesse BW, Croyle RT, Willis G, Arora NK, Rimer BK, Viswanath KV, Weinstein N, Alden S (2004). The Health Information National Trends Survey (HINTS): development, design, and dissemination. J Health Commun.

[34] (2017). Health Information National Trends Survey.

[35] Health Information National Trends Survey.

[36] Löwe B, Wahl I, Rose M, Spitzer C, Glaesmer H, Wingenfeld K, Schneider A, Brähler E (2010). A 4-item measure of depression and anxiety: validation and standardization of the Patient Health Questionnaire-4 (PHQ-4) in the general population. J Affect Disord.

[37] (2018). National Cancer Institute.

[38] Conci M, Pianesi F, Zancanaro M (2009). Useful, social and enjoyable: Mobile phone adoption by older people.

[39] Plaza I, Martín L, Martin S, Medrano C (2011). Mobile applications in an aging society: Status and trends. Journal of Systems and Software.

[40] (2018). Pew Research Center.

[41] Manafo E, Wong S (2012). Health literacy programs for older adults: a systematic literature review. Health Educ Res.

[42] Baumel A, Yom-Tov E (2018). Predicting user adherence to behavioral eHealth interventions in the real world: examining which aspects of intervention design matter most. Transl Behav Med.

